# Behavioural Factors Influencing the Intention to Adopt Sheep Scab Control Measures in Northern Ireland

**DOI:** 10.3390/ani14060912

**Published:** 2024-03-15

**Authors:** Adewale Henry Adenuga, Paul Crawford, Aurelie Aubry, Sharon Verner, Sam Strain, Stewart T. G. Burgess

**Affiliations:** 1 Economics Research Branch, Agri-Food and Biosciences Institute, Belfast BT9 5PX, UK; 2 Northern Ireland Sheep Scab Group, Islandmagee BT40 3RW, UK; 3 Livestock Production Sciences Branch, Agri-Food and Biosciences Institute, Large Park, Hillsborough BT26 6DR, UK; 4 Animal Health and Welfare Northern Ireland, Dungannon BT71 6JT, UK; 5 Department of Vaccines & Diagnostics, Moredun Research Institute, Pentlands Science Park, Bush Loan, Penicuik EH26 0PZ, UK

**Keywords:** ruminant, endemic disease, serological testing, theory of planned behaviour

## Abstract

**Simple Summary:**

Sheep scab, caused by infestation with the ectoparasitic mite *Psoroptes ovis*, is endemic in Northern Ireland (NI). The disease continues to cause NI’s sheep farming industry significant challenges, negatively impacting sheep and farmer welfare. The diagnosis of sheep scab has traditionally been achieved by taking skin scrape samples from suspected lesional areas on sheep suspected of being infested with sheep scab. These skin scrape samples are then examined under a microscope to confirm the presence of *Psoroptes ovis* mites, which provides a definitive diagnosis of the disease. However, while this method of diagnosis is highly specific for sheep scab, there can be a high level of variability in the sensitivity of the method, which can be as low as 18 percent. The objective of this study was to analyse the behavioural factors influencing the intention of sheep farmers to adopt sheep scab control measures in the form of blood testing. To achieve our objective, we analysed data from a sample of 126 sheep farmers using quantitative techniques. The results show that the promotion of a positive attitude towards blood testing for subclinical diagnoses of the disease would go a long way in controlling sheep scab in NI.

**Abstract:**

Sheep scab, caused by infestation with the ectoparasitic mite *Psoroptes ovis*, is an endemic disease in the Northern Ireland (NI) sheep flock and constitutes significant economic and welfare burdens for the NI farming industry. Despite its endemic nature, historically, little research has been undertaken to support the control of the disease in NI. This study offers the first attempt to analyse the psychological and behavioural factors influencing farmers’ intentions to implement effective sheep scab control measures in NI. To achieve our objective, quantitative data from a sample of 126 sheep farmers were statistically analysed using an extended theory of planned behaviour approach in an ordered logistic regression modelling framework. Our analyses showed that sheep scab remains an issue of concern in Northern Ireland. The attitudes of the farmers, as well as perceived behavioural control, emotional effect, membership of Business Development Groups (BDGs), and higher education qualifications, were found to be statistically significant factors influencing farmers’ intentions to adopt sheep scab control measures. This study provides a solid foundation for how to promote behavioural changes among sheep farmers to improve their ability to implement effective disease control measures, helping to tackle this challenging disease in a more sustainable way in the future.

## 1. Introduction

Sheep scab, caused by infestation with the ectoparasitic mite *Psoroptes ovis*, is endemic in Northern Ireland (NI) [1,2]. The disease is highly contagious, resulting in intense pruritus and significant economic concerns for the NI sheep farming industry [2]. In particular, the disease has a significant effect on the welfare of sheep, also likely having a consequential effect on farmers’ mental health [2,3]. This is reflected in the clinical signs, which include “severe itching and scratching, loss of wool, restlessness, biting at flanks, exudative and pruritic skin lesions, skin covered in scabs and thickened skin, skin excoriation and secondary skin infections, severe pain, weight loss (which can be severe), low birth weights and higher perinatal mortality rates in lambs born to affected ewes” [2]. In severe cases, the condition can be more extreme, resulting in emaciation, depression, and even death [2].

Northern Ireland has a national flock of approximately two million sheep which spread across 38% of the total number of farms in the region [4]. Farms with more than 500 sheep account for 41% of the total sheep population, and 90% of farms have at least 100 sheep [4]. An important part of farming in the region is the short-term land rental system, referred to as conacre, which is unique to the island of Ireland (Northern Ireland and the Republic of Ireland), in which land is rented nominally for 11 months or 364 days and there is no requirement for either party to enter into long-term agreements [5]. A previous study by Milne, et al. [6] has found that the highly fragmented but interconnected farming system resulting from the prevalence of the conacre system may contribute to the spread of disease between animals on contiguous land parcels.

It has been estimated that the cost of contracting sheep scab for a flock of 300 ewes could be as high as GBP 1000–GBP 2400 per outbreak for a lowland flock and GBP 1000–GBP 2100 per outbreak for an upland flock, with the cost influenced by the lambing ratio, the type of treatment used, and the time of the year [7]. These estimates are corroborated by a recent study in NI by Crawford, Hamer, Lovatt and Robinson [1], which made use of farmer-provided estimates following an actual outbreak. This study showed that the financial cost of sheep scab outbreaks can be as high as GBP 2500 per farm. The effective control of the disease is therefore essential, given its potential effect on the profitability and sustainability of sheep farms if not properly controlled.

Sheep scab is notifiable in NI under the Sheep Scab (NI) Order 1970 (as amended) [8]. This requires any suspicion of the disease to be reported to the Department of Agriculture, Environment and Rural Affairs (DAERA) [8]. The order places restrictions on the reporting flock until written confirmation is received from the flock’s Private Veterinary Practitioner (PVP) declaring that the flock has been correctly treated with a product licensed for the effective treatment of sheep scab. Despite this policy, the disease remains prevalent in NI [1]. The main objective of this study was to analyse the psychological and behavioural factors influencing farmers’ intentions to adopt sheep scab control measures in NI. The sheep scab control measures are defined in terms of farmers’ willingness to undertake serological testing to determine the flock’s disease status using a blood test (enzyme-linked immunosorbent assays (ELISAs)). The level of prevalence and the barriers to effective control of sheep scab were also assessed.

The diagnosis of sheep scab has traditionally been achieved by taking skin scraping samples from suspected lesional areas on sheep suspected of being infested with sheep scab. These skin scrape samples are then examined under a microscope to the confirm the presence of *P. ovis* mites, which provides a definitive diagnosis of the disease [2]. However, while this method of diagnosis is highly specific for sheep scab, there can be a high level of variability in the sensitivity of the method, which can be as low as 18 percent [2]. The implication of this is that sheep scab may not be confirmed until there is a very high level of clinical disease, which carries the associated risk of the disease spreading to other groups of sheep or neighbouring flocks [2]. Additionally, the clinical signs of sheep scab bear a common resemblance to that of a louse infestation, as both diseases result in intense pruritus, scratching, and wool loss, but they do not always require the same treatment method [2,9,10]. Therefore, relying on the presence of clinical signs or the identification of active skin lesions constitutes a potential hindrance to the effective control of sheep scab [1,10,11].

Serological testing for sheep scab is now available using an ELISA-based blood test. This is a very effective method of detecting the disease, with high levels of sensitivity and specificity, which is also capable of identifying an infestation prior to the advent of clinical signs, within 2 weeks of an infestation event [12]. The more widespread use of this test would allow for the early identification of cases, reducing the transmission of the disease and making it possible to develop targeted and coordinated treatments [2,13].

While some work has been undertaken in Great Britain in relation to sheep scab, there is a significant lack of information on the factors that influence the attitudes of farmers in relation to control strategies and barriers to optimum infection control. This study aims to fill this gap in the literature by improving the understanding of the disease in the NI flock and providing knowledge and evidence to inform the design of efficient and sustainable policies to control the disease through behavioural change. The study is also expected to have a significant welfare and social impact on the sheep farming industry through effective treatment selection given that outbreaks of scab in a flock often results in stress and hardship for both livestock and farmers.

## 2. Materials and Methods

To achieve the objective of this study, we first conducted a number of Focus Group Discussions (FGDs) with key stakeholders in the NI sheep farming industry to determine their views of sheep scab and to develop the survey questionnaire. The FGDs formed part of the wider project, which involved a self-reporting scheme for scab in which farmers and their private veterinary surgeons (PVS) could notify an active case of sheep scab. This involved the serological confirmation of reported cases, coordinated treatments, and the provision of best practice advice. The results of the FGDs are reported in [14]. The quantitative aspect involved the use of descriptive statistics and econometric techniques which combined an extended theory of planned behaviour (eTPB) model with Principal Component Analysis (PCA) and ordered logit regression models [15].

### 2.1. Questionnaire Design and Survey Development

To design the questionnaire used for the quantitative analysis, information was obtained from FGDs and a comprehensive review of the literature about sheep scab and related diseases. The questions were organised around several themes to capture the variables required for analyses. These included the farmers’ experiences with sheep scab, socioeconomic characteristics, and behavioural and attitudinal questions. For some questions, the farmers were able to select more than one option in their response. The questionnaire was developed using a hybrid approach and comprised mainly closed-ended questions. The farmers were, as a result, able to complete the questionnaires either online or on paper. The Snap XMP survey software version 12.15 [16] was used to develop the online version of the questionnaire. A QR code was placed on the front page of the paper questionnaire so it could be easily scanned by the respondents using a mobile phone. The questionnaire also involved an open-ended question in which farmers were asked to state their experience with sheep scab in NI. The survey period took place between 25 November 2022 and 19 May 2023. A reminder was also sent during this period to each participant. It took approximately 20 min to complete the questionnaire, and the data were cleaned and checked for outliers before analysis was undertaken. The survey was anonymous, with no personal information being included in the questionnaire. The farmers were encouraged to complete the questionnaire by providing them with an opportunity to enter a prize draw for 1 of 5 GBP 100 e-vouchers. This required them to provide their contact details, which were stored separately from the survey responses and only used to contact the winners of the draw.

### 2.2. Study Sample and Data Collection

The sampling frame was the census data for NI sheep farmers [4]. We randomly selected 600 farmers, who, based on their return to DAERA’s annual sheep inventory, had at least 100 sheep in their flock to whom we administered our questionnaires. A further 100 questionnaires were reserved and administered to the sheep farmers who participated in the support scheme [14]. In total, we received 126 responses, out of which 21 were from the support scheme’s participants. Overall, 90 of the questionnaires were completed on paper, while 36 were completed online. There were some missing data points, as some of the farmers did not completely fill out the questionnaire, and two farmer’s responses were removed due to the small number of questions completed. For the econometric analysis, a further nine observations were removed due to ambiguous responses and missing data points.

### 2.3. Statistical Analyses

Statistical analyses were undertaken in three stages. In the first stage, descriptive statistics were used to analyse the farmers’ socioeconomic characteristics and the direct measures of the eTPB constructs. The second stage involved the use of PCA to ensure that the items that were loaded on the individual eTPB hypothetical constructs were consistent [17]. A previous study conducted by Kautonen, et al. [18] employed a similar approach to validate TPB constructs. In the third and final stage, an ordered logistic regression model was used to analyse the associations between behaviour-specific baseline eTPB constructs and the sheep farmers’ intentions to implement sheep scab control measures.

#### 2.3.1. The Theory of Planned Behaviour

To capture the sheep farmers’ behaviour towards adopting sheep scab control measures, we employed an extended theory of planned behaviour (eTPB) model. It is widely acknowledged in the literature that farmers’ behaviour and decision making take place in a dynamic and complex environment, being potentially influenced by several factors, including social, economic, political, and ecological factors [19]. The TPB stipulates that intention to perform a behaviour is jointly determined by three psychological constructs: attitude (ATT), subjective norm (SN), and perceived behavioural control (PBC) [20]. Attitude, by definition, refers to the evaluation of the behaviour of interest in terms of how it is being viewed as either favourable or unfavourable. Subjective norm, on the other hand, refers to perceived social pressure towards the behaviour, while PBC is the personal assessment of the feasibility of executing the behaviour in a given context [21,22]. In this study, the behaviour of farmers in relation to implementing sheep scab control measures was analysed using an extended TPB model with the inclusion of an additional construct of emotional effect (EE). Previous studies have shown that extending the TPB increases its predictive power [23].

It was hypothesised that the decision of a sheep farmer to undertake blood testing for sheep scab is influenced by the farmer’s attitude (ATT) towards sheep scab control measures; perceived pressure from significant others (SN) such as other farmers, vets, family members, etc.; farmers’ perception of their ability to implement sheep scab control measures (PBC); and the emotional effect (EE) that can result from the presence of sheep scab in the flock. Statements reflecting the constructs of the extended TPB were developed and used as measurement indicators. The statements were measured using a 5-point Likert scale anchored in the extreme points to capture the intention to implement sheep scab control measures, ATT, SN, PBC, and EE, with lower values (1 and 2) standing for “strongly disagree and disagree” and high values (4 and 5) standing for “strongly agree and agree”, while the middle number (3) was interpreted as a neutral response. Previous behavioural studies, for example, that of Tensi, et al. [24], have also used a 5-point Likert scale. Where necessary, items were scored in reverse in a way, meaning that variables with a high score represented an increased quantity of the measure.

#### 2.3.2. Ordered Logistic Regression Model

The ordered logistic regression (OLR) model was used in this study to analyse the behavioural and socioeconomic factors influencing the likelihood of adopting blood testing for the rapid and accurate diagnosis of sheep scab [25]. In the first instance, we considered only behavioural factors (ATT, PBC, SN, and EE) influencing the decision to undertake the blood test and their willingness to pay for the blood test in our OLR models. We then subsequently undertook analyses with the inclusion of socioeconomic factors. The inclusion of the covariates in the model helped to adjust for potential imbalances in the baseline variables that may be related to the outcome of interest [26]. The ordered logit regression model is specified in Equation (1).

(1) $$\mathrm{Pr}\left(Y_{i}\right)>j=\frac{\exp(\alpha_{j}+X_{i}\beta )}{1+\left\{\exp\left(\alpha_{j}+X_{i}\beta \right)\right\}}\;,\;\;\;\;\;\;\;\;\;\;\;\;\;j=1,\;2,\;\ldots ,M-1$$

where *j* is the response category, $X_{i}$ is a vector of explanatory variables, β is a vector of parameters to be estimated, and $\alpha_{j}$ refers to the cut-off points for the thresholds of the ordered model, and the number of categories of the ordinal response variable is represented by *M*. Although the results of the ordered logit model are usually easy to interpret, there is the limitation of the parallel lines assumption, which assumes that the value of β is the same for each *j*. Violation of the parallel lines assumption may result in incomplete interpretations of the study results [27]. The Brant test is usually used to check whether the parallel lines assumption has been violated [28]. The generalised ordered logit regression model can overcome the limitation of the parallel lines assumption by relaxing it [27,29]. However, there are usually too many parameters to be explained compared to the ordered logit model. The partial proportional odds model is regarded as a middle ground between the generalised ordered logit model and the ordered logit model, as it constrains the variables that violate the parallel lines assumption to the same coefficients [29]. The use of both the generalised ordered logit model and the partial proportional odds model is only necessary if the parallel lines assumption is violated. Otherwise, the ordered logit regression model is employed. In addition to the behavioural factors’ variables of attitude, perceived behavioural factors, subjective norm, and emotional effect, the socioeconomic factors included in the model are the age of the farmer, membership of a business development group (BDG), time commitment to farming (whether full-time or part-time), and level of agricultural and formal educational qualifications.

## 3. Results

### 3.1. Socioeconomic Characteristics

An overview of the socioeconomic and farm characteristics of the respondents is presented in Table 1. The denominator used reflects the number of farmers who responded to the specific question. The results show that about 52% of the sheep farmers in our sample were at least 54 years old, and the modal age group was 41 to 54 years. The majority of the respondents were male (92%), and 27% had no formal educational qualification. For 58% of the respondents, farming is something they undertake on a full-time basis. Based on flock type, about half (51%) of the farmers can be categorised as lowland sheep farmers. Overall, 56% of the sheep farmers surveyed reported that they had off-farm income, while only 14% reported that they had diversified activities on their farm. The average number of breeding ewes was 174, and the average farm size was 56.2 hectares. The average years of farming experience was relatively high at 33 years, and only 38% of the farmers reported that they had definitely identified a successor. In addition to keeping sheep, 25% of the respondents had a beef finishing enterprise, 44% had a beef suckler enterprise, and 5% had dairy and poultry enterprises, respectively, while 4% had an arable enterprise, and 8% had beef finishing and suckler enterprises. Thirty-four percent of the respondents reported having only a sheep enterprise. For the majority of the farmers, the main product from their flock was finished lamb (53%), followed by store lambs (23%) and breeding females (8%). The remaining farmers (16%) produce a combination of any of these three products.

### 3.2. Reported Incidence of Sheep Scab

Table 2 provides a summary of the reported incidence of sheep scab among the sheep farmers. The results show that 32.2% of the farmers had experienced sheep scab on their farm at least once between 2012 and 2022. Only 37.5% of the farmers who had experienced sheep scab on their farms reported that the diagnosis was confirmed by a veterinarian. Nine percent of farmers in our sample reported that they knew nothing about sheep scab. A total of 36% of the respondents with scab incidence on their farm stated that the diagnosis was confirmed by a vet, while 18% stated that they had experienced scab at least twice in the last 10 years.

**Figure 1:**
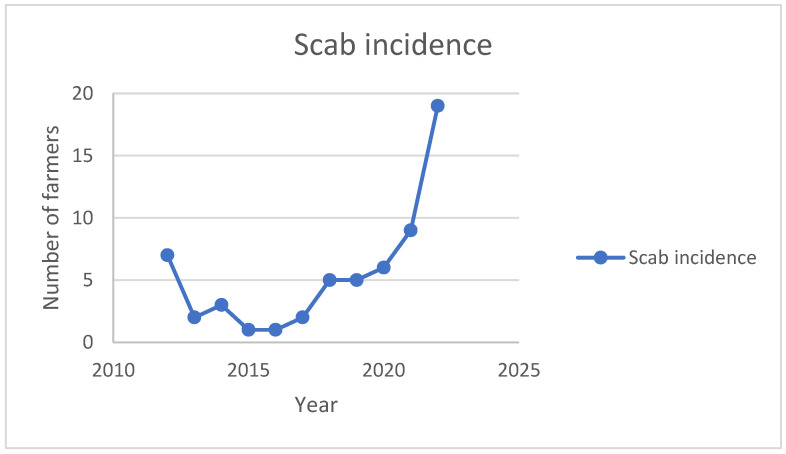
Reported incidence of sheep scab (2012 to 2022) among surveyed farmers.

Figure 1 shows the trend of the reported incidence of sheep scab among the sheep farmers who stated that they had had scab on their farms between 2012 and 2022. The results show that there has been an increase in the number of reports of the disease in recent years, with the highest reporting occurring in 2022 (46%).

Seventy-six percent of the farmers in our sample reported that “bought-in animals” were quarantined on arrival, and the modal quarantine period was three to four weeks. Among the farmers that had experienced scab in their flock, 77% reported that all bought-in animals were quarantined on arrival on the farm, with a modal quarantine period of three to four weeks. This implies relatively equal level of quarantine procedure for the overall sample and for the sub-sample of farmers that had experienced scab on their farms. However, 33.3% of the farmers stated that they quarantined their new animals for less than the recommended three weeks [1].

From the results of the open-ended question in our survey, the market was identified as a high-risk place that occupies a pivotal position in the transmission of sheep scab. In the words of one of the farmers:

*“In my opinion sheep scab has become more prevalent. I buy in store lamb and find that most sheep I bought have not been treated. This is the source of the infection. Not enough farmers are adequately controlling it”*.

### 3.3. Potential Barriers to Sheep Scab Control

We analysed potential barriers to the uptake of sheep scab control measures. The farmers were asked to state their level of agreement to selected statements that could be considered as a potential hindrance to the control of sheep scab using a 5-point Likert scale of strongly disagree (1); disagree (2); neutral (3); agree (4); and strongly agree (5). The results are presented in Table 3. The results showed a relatively high reliance on the presence of clinical signs to rule out the possibility that newly purchased sheep were infested with sheep scab.

### 3.4. Sheep Scab Diagnosis among Farmers That Had Experienced Sheep Scab

**Figure 2:**
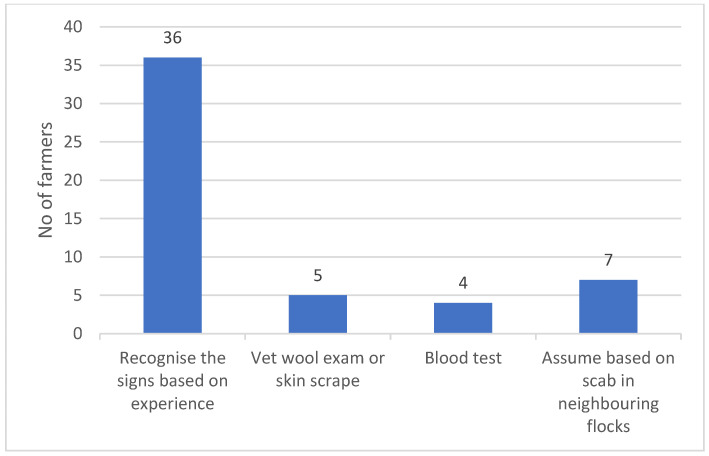
Method of diagnosis of survey respondents that had experienced sheep scab between 2021 and 2022.

The distribution of how the diagnosis of scab was reached by the farmers is presented in Figure 2. Of the farmers who had experienced sheep scab, only 10% reported using blood testing for diagnosis. The majority of the farmers, (90%) reported that they recognised the signs based on experience. About 12.5% reported using their private vet to collect and examine wool samples or skin scrapes, and 17.5% reported confirming sheep scab based on their neighbouring flocks’ status.

### 3.5. Results of Econometric Analysis

#### 3.5.1. Principal Component Analysis (PCA)

The results of the PCA analysis are presented in Table A1 in Appendix A. The PCA’s four components were obtained from 16 attitudinal statements. The Kaiser–Meyer–Olkin measure of sampling adequacy was 0.67, which implies that the four components represent a significant proportion of the variance in the data [26]. In line with normal practice, components that had an eigenvalue of at least one were retained. The application of a promax rotation was necessary to explain the components’ field [15]. Statements that had loadings greater than or equal to 0.3 on their target factor were retained. Overall, we found that the four components explained 48% of the total variance of the original variables. It has been suggested in the literature that around 50% of the explained variance of the original data set can be regarded as adequate [26]. The internal consistency, validity, and reliability of the set of items for each latent construct in the model were analysed using Cronbach’s alpha (α) coefficient. A construct is considered to demonstrate adequate reliability if the alpha coefficient is 0.6 or above [30]. The Cronbach’s alpha coefficients for all the constructs showed good-to-excellent reliability, ranging from 0.64 to 0.86.

#### 3.5.2. Measurements of Items in the Theory of Planned Behaviour (TPB) Models

A summary of the farmers’ responses to the measurement items, reliability coefficients, means, and standard deviations (SDs) for the eTPB model are presented in Table A2 in Appendix A. The attitude of the sheep farmers to the implementation of sheep scab control measures was assessed using four statements after conducting the PCA. In general, the farmers showed a positive attitude toward sheep scab control measures, with a mean value of 4.21 for all items used to measure the construct. The farmers generally agreed that sheep scab control measures were worth implementing and that implementing sheep scab control measures has the potential to improve the productivity and welfare of their flock. The average score for all the items used in measuring the perceived behavioural construct was 3.8. The result indicates that farmers generally felt that they were able to identify sheep scab and had the skills needed to control it.

#### 3.5.3. Results of Ordered Logit Model

The results of the Ordered Logit Model are presented in Table 4. A Brant test was undertaken to test whether the parallel lines assumption was violated. The result showed that it was not statistically significant, indicating that the null hypothesis of the parallel lines assumption had not been violated (using a threshold of *p* < 0.1) for Model 1 (chi-square = 11.98; *p*-value = 0.447) and Model 2 (chi-square = 11.03; *p*-value = 0.526). The results for goodness-of-fit, tested using the Hosmer–Lemeshow test [31]—Model 1 = (likelihood ratio statistic = 61.08, degree of freedom = 55, *p*-value = 0.267); Model 2 = (likelihood ratio statistic = 58.60, degree of freedom = 55, *p*-value = 0.3447)—also revealed that the models fitted well. As with other logistic regression models, the coefficients for the ordered logit model are not directly interpretable [17]. Holding all other variables constant, in this study, we interpreted the coefficients as standardised changes in odds for a unit change in the explanatory variable [29]. This was carried out using the listcoef post-estimation command implemented in the Stata package SPost13 [32].

The results show that ATT, PBC, and EE were statistically significant at the 5% level for factors influencing the intention to undertake blood testing. A standard deviation increase in the ATT factor increases the odds of the average farmer undertaking blood testing by 52%. The EE and PBC factors have a negative relationship with intention to undertake blood testing. This implies that, for example, a standard deviation increase in the EE factor decreases the odds of embracing the use of blood testing by 36%. On the other hand, attitude and emotional effect were statistically significant at the 1% and 10% levels, respectively, for factors influencing willingness to pay for blood testing.

Analyses were also conducted following the addition of socioeconomic variables to the models, and the results are presented in Table 5. The results show that taking into account socioeconomic variables also led to ATT and EE being statistically significant at the 1% and 10% level, respectively, for the intention to undertake blood test analysis. Specifically, a standard deviation increase in the ATT factor increases the odds of the average farmer undertaking blood testing by 97%. On the other hand, EE was found to have a negative association with the intention to undertake blood testing. A standard deviation increase in the EE reduces the odds of the average farmer undertaking blood testing by 28%. Perceived behavioural control was no longer statistically significant, and SN was also not associated with the intention to undertake the blood testing. Membership of a Business Development Group (BDG) and the attainment of A Level–Higher education qualifications or equivalents were also statistically significant factors (at the 5% level) influencing the intention to undertake blood testing. A standard deviation increase in the BDG membership factor increases the odds of the average farmer undertaking blood testing by 72%, while a standard deviation increases in the attainment of A Level–Higher education factor increases the odds of the average farmer undertaking blood testing by 75%. In terms of willingness to pay for blood testing, ATT remained statistically significant at the 1% level, but EE was no longer statistically significant, although the relationship remained negative. In terms of socioeconomic characteristics, the age of the farmers was the only statistically significant factor at the 5% level for the factors influencing willingness to pay for blood testing. All other socioeconomic factors were not statistically significant. We also ran the analyses after excluding the non-significant variables from the models. All the variables remained statistically significant, except for the emotional effect variable, for the intention to pay for blood testing. This is for the model without the socioeconomic variables. However, the signs remain the same. This reflects the initial marginal level of statistical significance (*p* < 0.1) of the variable. The results are available in the Supplementary Materials.

**Figure A1:**
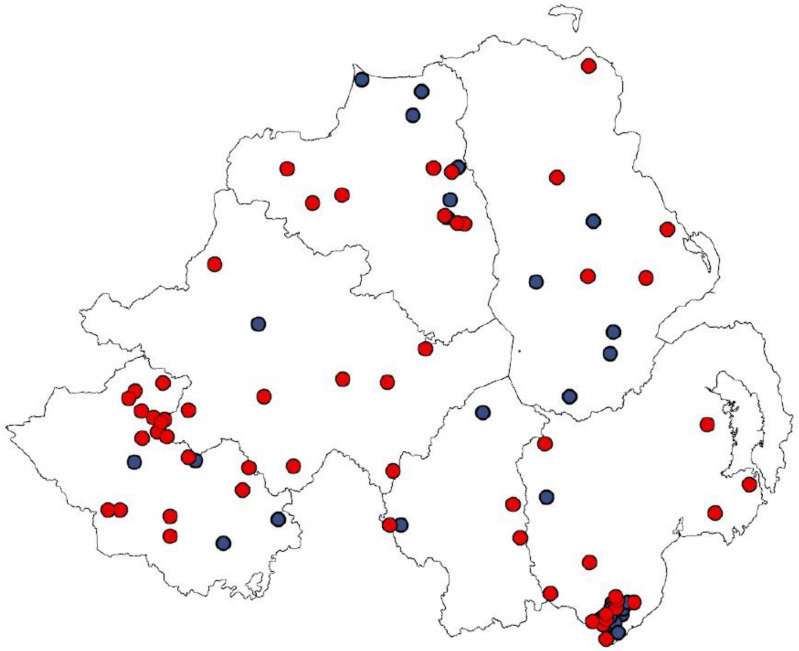
Map showing geographical location of participating farmers, including common graziers. *Key: Red dots—scab-positive flocks. Blue dots—no confirmation of scab*.

As part of this study, a scheme which allowed the farmers to report to their local vet or the project managers if they suspected any scab infestation in their flock was developed. The farmers who participated in the scheme were offered on-farm consultations, sample collection and analysis support, and treatment support. Figure A1 in Appendix B gives the distribution of scab in Northern Ireland for the 105 farmers that participated in the scheme. The results show that scab is widely distributed geographically across the six counties in NI.

## 4. Discussion

The results of our analysis showed that sheep scab remains an issue of concern in Northern Ireland. This may partly be associated with the increased awareness of the disease brought about by the Sheep scab NI group and the self-reporting scheme/workshops related to this project held in 2022. Our results show that there is still a high reliance on the presence of clinical signs for the diagnosis of sheep scab in the region. This may be a significant barrier to the control of sheep scab as the presence of subclinical disease could easily be missed. Three other important factors that seem to hinder the effective control of sheep scab are the cooperation among farmers to control the disease, the prevalence of the short-term (conacre) land rental system, and the lack of adequate training among farmers on the effective control of sheep scab. A good percentage of our respondents (27%) agreed or strongly agreed that they find it difficult to get neighbours to cooperate with the control of sheep scab and other animal diseases. The fact that close to a third (31%) of the farmers stated that they do not have adequate training regarding sheep scab control implies that more awareness and training regarding the best practices to control of the disease are required to improve the control of sheep scab and echoes the findings of [1].

The accurate and timely diagnosis of sheep scab is essential to ensure the appropriate use of treatments, which are heavily relied upon for the effective control of the disease [2]. The results showed that there is still a low level of awareness regarding the subclinical phase of sheep scab. Studies have shown that traditional methods of sheep scab diagnosis have low sensitivities and are often unable to determine subclinical infestations [1,10,12]. The coordinated use of reliable diagnostic tests such as sheep scab blood tests is therefore needed to identify subclinical infestations and facilitate the targeted and coordinated control of the disease. The level of sensitivity and specificity of the blood tests are high, at 98% and 97%, respectively, and crucially, blood testing is able to detect sheep scab at the flock level from as early as two weeks post-infestation and before the appearance of clinical signs [10,12]. The blood tests are commercially available and have the potential to contribute immensely to the control of sheep scab through specific, rapid, and accurate diagnoses of the disease [1].

For the econometric analysis, the attitude of the farmers had the greatest impact on their intention to undertake and pay for blood testing. This is a significant result, as it demonstrates how much a change in attitude of the farmers can positively influence sheep scab control measures. In addition, the negative and statistically significant impact of the emotional effect in the intention to undertake blood test model shows that farmers who are emotionally affected by the presence of sheep scab in their farms are less likely to want to seek a new approach to control the disease. We find this to be an interesting result, as we had expected that the emotional effect would indicate that farmers care about it and thus are keen to get rid of it. However, it seems that the emotional effect here relates more to shame and a feeling of helplessness. This is reflected in the result in which we found that 55% of the farmers either agreed or strongly agreed to the statement saying that “Having sheep scab in my flock can make me feel miserable”. This may also imply that the farmers are less likely to want to share information about the presence of scab in their farms with other farmers, which could contribute to the spread of the disease. A previous study by Crawford, Hamer, Lovatt and Robinson [1] also reported the under-reporting of the disease by sheep farmers in Northern Ireland. A farmer in one of the open-text responses in our survey stated the following:

*“No one wants to share the knowledge that they have sheep scab as neighbours would think it was bad shepherding and management”.* Our results are in line with those of Crawford, Hamer, Lovatt and Robinson [1], who showed that a sheep scab outbreak can have not only economic costs but also an emotional cost for farmers, which could affect their level of decision making. Perceived behavioural control was also found to be negative and statistically significant factor influencing the intention to undertake blood testing in the model without the socioeconomic variables. It was, however, not statistically significant for willingness to pay for blood testing. Perceived behavioural control is an indicator of the extent to which the farmers believe they understand how to control the disease. The negative relationship implies that farmers believe that they know enough about scab, making them less interested in undertaking blood testing. However, having an erroneous confidence about the disease could contribute to its spread. There is a need to provide more information to farmers on the importance and significance of undertaking blood test to control scab.

In contrast, the education of the farmer and whether they are a member of a BDG are important factors that could have a positive influence on farmers’ decision to undertake blood testing. The BDGs are ‘peer-to-peer’ learning farmer groups that were established in Northern Ireland in March 2016. The scheme brings farmers together under the guidance of a facilitator to share knowledge and help them improve their technical efficiency and business management skills [33]. Farmers who are members of a BDG and those who are educated are more likely to undertake blood testing. Farmers who are educated are more likely to undertake blood testing compared to farmers with no education. This relationship was confirmed when the analysis was undertaken considering only the statistically significant variables. Devising programmes that are aimed at getting farmers to obtain educational qualifications, for example, through evening classes, have the potential to help enhance the control of the disease. While age was not statistically significant for intention to undertake blood testing, our results show that it was a positive and statistically significant factor influencing the willingness to pay for blood test. This result implies that younger farmers (less than 55 years) are more likely to pay for blood testing compared to older farmers. This is understandable, as younger farmers are likely to be more inclined to innovation compared to older farmers. This points to the need to encourage the younger generation to become more engaged in the farming business.

The market was identified by the farmers in the survey’s open-ended question as a high-risk place for the spread of sheep scab. It is essential that appropriate measures are put in place to control the spread of the disease through markets. A previous study by Smith, Ruston, Doidge, Lovatt and Kaler [11] also found similar results.

## 5. Conclusions and Policy Recommendations

To develop policies capable of driving change in relation to the control of scab, it is essential to understand the behavioural factors influencing farmers’ decisions to implement best practice control measures. This study has provided novel insights into the prevalence of sheep scab in Northern Ireland and has also analysed the behavioural factors that could influence farmers’ intentions to implement a sheep scab control measure (blood testing).

Given the significant influence of farmers’ attitude on their intention to undertake and pay for blood testing as a way of diagnosing sheep scab, any intervention aimed at controlling sheep scab will benefit from the promotion of positive attitudes towards control measures. This can be achieved through individually tailored messages to address the underlying factors influencing such attitudes, for example, through increasing the awareness of the significant detrimental impact of the disease on animal welfare and production. Sheep farmers’ attitudes towards sheep scab control measures could also be influenced by the incorporation of animal welfare topics in the BDG programme and the inclusion of local vets as facilitators. There is a need for an increased awareness of the disease and its effect, particularly to help farmers feel more comfortable about reporting the disease without feeling ashamed by having it diagnosed in their flock. Support for farmers in the form of helping with diagnosis and treatment costs could help reduce the emotional pressure resulting from the diagnosis of the disease on their farms. Although, farmers may be uncomfortable with the restriction often placed on their farms in the event of a sheep scab diagnosis, there is a need for more interaction between the Department of Agriculture, Environment and Rural Affairs and the farmers on this policy. Given that about 27% of farmers in our sample either agreed or strongly agreed that they find it difficult to get neighbours to cooperate in the control of sheep scab and other animal diseases, it is essential that a strategy is put in place to ensure a coordinated effort in the control of the disease. This will involve highlighting the significance of cooperation to the farmers. Success in the control of the disease can never be achieved in isolation but rather through cooperation between farmers in affected cluster areas. There is a clear need for stricter measures to ensure that only clean and healthy stock is brought to the market for sale to prevent the spread of the disease through this route. There is also a need for the expansion of the awareness of the disease and training programmes regarding best practice treatment and control measures for sheep scab. For example, farmers should be encouraged to use OP dipping rather than relying on injectable MLs, for which resistance has now been reported in sheep scab mites, and they are also heavily relied upon for the control of gastrointestinal nematodes. In addition, the application of OP dipping via showers or jetters should be discouraged, as it is not an effective means of delivering these crucial treatments and will undoubtedly contribute to the development of OP resistance in the future. A lack of efficient quarantining for purchased animals poses a significant risk of bringing the disease onto the farm. An increased awareness of the risks involved, and effective mitigation strategies are clearly required. While this study was undertaken in relation to sheep scab, the findings apply to the control of other endemic diseases for which blood testing is an option. Therefore, further research focusing on other endemic diseases of livestock would be valuable. A possible limitation of this study is the bias that may have arisen from social desirability, as farmers may have overestimated their positive disposition towards blood testing as they may have felt it might provide an opportunity to secure grant funding from the government. However, it is our belief that such bias must have been reduced by the clear explanation of the objectives of the survey provided to the farmers.

## Figures and Tables

**Table 1:** Socioeconomic characteristics of survey respondents.

Variables	Frequency	Percentage (%)
Flock type		
Hill	28	22.2
Lowland	63	50.8
Upland	33	26.6
Time Commitment (*n* = 124)		
Farming full-time	72	58.1
Farming part-time	52	41.9
Off-farm income (*n* = 124)		
Yes	69	55.6
No	55	44.4
Educational Qualification (*n* = 124)		
Degree-level or higher	26	22.0
Higher education—diploma or equivalent	19	15.3
A-level or equivalent	11	8.9
5 GCSEs or equivalent	20	16.1
Less than 5 GCSEs	15	12.1
No formal qualifications	33	26.6
Presence of a successor (*n* = 123)		
Yes, a successor has been identified	47	38.2
Likely, but still not certain	30	24.4
Uncertain, too early to say	32	26.0
No, definitely not	14	11.4
Diversified Activities (*n* = 124)		
Yes	17	13.7
No	107	86.3
Membership of BDG (*n* = 124)		
Yes	83	66.9
No	41	33.1
Gender (*n* = 124)		
Male	114	91.9
Female	9	7.3
Prefer not to say	1	0.8
Marital Status (*n* = 124)		
Single, never married	13	10.5
Married	98	79.0
Civil Partnership	3	2.4
Separated	4	3.2
Divorced	2	1.6
Widowed	4	3.2
Age (*n* = 124)		
Less than 30	9	7.3
Age 30–40	15	12.1
Age 41–54	40	32.3
Age 55–64	28	22.6
Age 65–74	24	19.4
Age 75 plus	8	6.5

**Table 2:** Reported incidence of sheep scab among surveyed farmers.

Incidence of Scab	Frequency	Percentage (%)
Yes	40	32.3
No	84	67.7
Confirmation of Diagnosis by a Vet		
Yes	15	37.5
No	25	62.5
Extent of Presence in Flock		
Only in one grazing group	21	52.5
In more than one grazing group	10	25.0
In all grazing groups	9	22.5
Severity of Scab Incidence		
No symptoms seen	1	2.5
Mild symptoms seen	16	40.0
Moderate symptoms seen	17	42.5
Severe symptoms seen	6	15.0

**Table 3:** Potential barriers to sheep scab control.

	Number (%) of Farmers Selecting Each Option					Mean	SD
Statements	SD (1)	D (2)	N (3)	A (4)	SA (5)		
I rely only on clinical signs to rule out the possibility that newly purchased sheep are infested with sheep scab before mixing them with their flock	15 (12.4)	22 (18.2)	13 (10.7)	65 (53.7)	6 (5.0)	3.20	1.17
I find it difficult to get neighbours to cooperate on the control of sheep scab and other animal disease	13 (10.8)	31 (25.8)	44 (36.7)	21 (17.5)	11 (9.2)	2.88	1.10
The conacre land rental system makes it difficult for me to implement sheep scab control measures	16 (13.5)	33 (27.7)	42 (35.3)	24 (20.2)	4 (3.4)	2.72	1.04
I do not have adequate training around sheep scab control	23 (19.2)	31 (25.8)	29 (24.2)	32 (26.7)	5 (4.2)	2.71	1.18
I find it difficult to get licensed personnel to carry out dipping	15 (12.7)	43 (36.4)	40 (33.9)	16 (13.6)	4 (3.4)	2.58	0.99
The control of sheep scab in my flock is physically demanding for me	22 (18.6)	42 (35.6)	29 (24.6)	18 (15.3)	7 (5.9)	2.54	1.14
I do not have an assistant to help with farm management and it is difficult to get a helping hand	23 (19.2)	42 (35.0)	28 (23.3)	21 (17.5)	6 (5.80)	2.54	1.14
The costs of controlling sheep scab are too much for me	31 (25.6)	41 (33.9)	33 (27.3)	9 (7.4)	7 (5.8)	2.33	1.11
Trying to control sheep scab takes up too much of my time	31 (26.1)	52 (43.7)	17 (14.3)	16 (13.5)	3 (2.5)	2.22	1.06

Note: SD = strongly disagree; D = disagree; N = neutral; A = agree; SA = strongly agree.

**Table 4:** Estimates of OLOGIT model for eTPB constructs.

	Intention to Undertake Blood Test				Intention to Pay for Blood Test			
Variables	Coef.	Std. Err.	%	%StdX	Coef.	Std. Err.	%	%StdX
Attitude (ATT)	0.184 **	0.078	20.1	52.1	0.213 ***	0.076	23.8	62.7
Perceived behavioural control (PBC)	−0.197 **	0.100	−17.9	−30.7	−0.041	0.090	−4.0	−7.3
Subjective norm (SN)	0.102	0.130	10.8	15.2	0.186	0.129	20.5	29.4
Emotional effect	−0.299 **	0.121	−25.9	−36.9	−0.186 *	0.112	−17.0	−24.9

Note: % is the percentage change in odds for a unit increase in the explanatory variable; %StdX is the percentage change in odds for a standard deviation change in the explanatory variable; single, double, and triple asterisks (*, **, ***) indicate significance at the 10%, 5%, and 1% levels, respectively.

**Table 5:** Estimates of OLOGIT model with adjustment for sociodemographic factors.

	Undertake Blood Testing				Willingness to Pay for Blood Testing			
Variables	Coef.	Std. Err.	%	%StdX	Coef.	Std. Err.	%	%StdX
Attitude (ATT)	0.296 ***	0.090	34.5	96.7	0.295 ***	0.089	34.3	96.3
Perceived behavioural control (PBC)	−0.036	0.112	−3.5	−6.4	0.009	0.106	0.9	1.7
Subjective norm (SN)	0.186	0.143	20.4	29.3	0.141	0.138	15.2	21.6
Emotional effect	−0.214 *	0.126	−19.3	−28.1	−0.174	0.119	−16.0	−23.5
BDG membership	1.128 **	0.465	208.9	71.5	0.132	0.435	14.1	6.5
Age (less than 55 years)	0.758	0.496	113.5	46.2	1.200 **	0.470	232.0	82.5
Full-time	0.186	0.482	20.4	9.7	0.531	0.442	70.1	30.1
Have formal agricultural qualification	−0.099	0.478	−9.4	−4.9	−0.522	0.464	−40.7	−23.0
Herd size (breeding ewes)	0.000	0.001	0.0	3.8	−0.002	0.001	−0.2	−27.9
Less than or 5 GCSEs or equivalent	0.900	0.567	145.8	50.5	0.101	0.537	10.6	4.7
A Level–Higher education or equivalent	1.313 **	0.619	271.7	74.9	0.251	0.595	28.5	11.3
Degree-level or higher	1.016	0.694	176.3	53.3	0.348	0.693	41.6	15.7

Note: % is the percentage change in odds for unit increase in the explanatory variable; %StdX is the percentage change in odds for a standard deviation change in the explanatory variable; single, double, and triple asterisks (*, **, ***) indicate significance at the 10%, 5%, and 1% level, respectively.

## Data Availability

The data that support the findings of this study were provided under a data sharing agreement with the Department of Agriculture, Environment and Rural Affairs (DAERA) and are not publicly available.

## Supplementary Materials

The following supporting information can be downloaded at: https://www.mdpi.com/article/10.3390/ani14060912/s1, Table S1: Estimates of OLOGIT model for eTPB constructs with only statistically significant variables. Table S2. Estimates of OLOGIT model with adjustment for socio-demographic factors with only statistically significant variables.

## Appendix A

**Table A1:** Principal components (component loadings) for eTPB constructs (values > 0.3 are highlighted in bold).

Variables	Attitude	PBC	Subjective Norm	Emotions
	PC 1	PC 2	PC 3	PC 4
**Attitude**				
There is no point implementing sheep scab control measures on my farm	**0.3104**	−0.0803	−0.0270	0.0617
I think sheep scab control measures are worth implementing	**0.3990**	0.0021	0.0108	0.0509
Implementing sheep scab control measures will improve the welfare of my flock	**0.4087**	0.0527	−0.0241	0.0664
Implementing sheep scab control measures will improve the productivity of my flock	**0.4135**	0.0690	−0.0456	0.0147
**Perceived behavioural control**				
I have a clear understanding of sheep scab	0.0755	**0.4291**	−0.0321	0.0545
I have the skills to identify the signs of sheep scab	0.0194	**0.4493**	0.0345	0.0887
I have the skills needed to control sheep scab in my flock	0.0346	**0.4530**	−0.0157	−0.0243
It is easy for me to detect sheep scab based on visible signs	−0.0760	**0.4090**	0.1135	0.0319
**Subjective norm**				
The opinion of DAERA is important to me in relation to the implementation of sheep scab control measures on my farm	0.0749	−0.1200	**0.3471**	0.1635
The opinion of my customers is important to me in relation to the implementation of sheep scab control measures on my farm	0.0847	−0.0122	**0.3739**	0.0454
The opinion of other farmers is important to me in relation to the implementation of sheep scab control measures on my farm	−0.1879	0.0452	**0.4472**	0.1118
The opinion of family members is important to me in relation to the implementation of sheep scab control measures on my farm	−0.0060	0.0637	**0.4143**	0.0398
**Emotions**				
Sheep scab is a disease that makes my life more difficult	0.0917	0.0260	−0.0442	**0.3546**
I feel ashamed when my flock has sheep scab	−0.0295	0.0265	0.0984	**0.4875**
I get frustrated with having to live with sheep scab in my flock	0.0461	0.0506	0.0686	**0.4732**
Having sheep scab in my flock can make me feel miserable.	0.1082	0.0399	−0.0859	**0.4488**

**Table A2:** Survey questions, reliability coefficients, means, and standard deviations (SDs) for the eTPB model.

Measurement Items	N	Mean	SD
Scab Control Measure (α = 0.73)	122	3.56	0.93
I am open to making use of blood test for the quick diagnostic of sheep scab on my farm before any visible signs	122	3.97	0.83
I am willing and ready to bear the costs of blood test for the quick diagnostic of sheep scab on my farm before any visible signs	121	3.12	1.22
Attitude (α = 0.85)	123	4.21	0.66
here is no point implementing sheep scab control measures on my farm	122	4.0	0.97
I think sheep scab control measures are worth implementing	122	4.3	0.68
Implementing sheep scab control measures will improve the welfare of my flock	123	4.3	0.73
Implementing sheep scab control measures will improve the productivity of my flock	122	4.2	0.78
Perceived Behavioural Control (α = 0.86)	124	3.7	0.74
I have a clear understanding of sheep scab	124	3.7	0.93
I have the skills to identify the signs of sheep scab	123	3.8	0.82
I have the skills needed to control sheep scab in my flock	124	3.8	0.77
It is easy for me to detect sheep scab based on visible signs	123	3.5	0.98
Subjective Norm (α = 0.64)	121	3.8	0.57
The opinion of DAERA is important to me in relation to the implementation of sheep scab control measures on my farm	121	3.6	0.90
The opinion of my customers is important to me in relation to the implementation of sheep scab control measures on my farm	119	4.0	0.74
The opinion of other farmers is important to me in relation to the implementation of sheep scab control measures on my farm	120	3.8	0.86
The opinion of family members is important to me in relation to the implementation of sheep scab control measures on my farm	120	3.9	0.77
Emotional effect (α = 0.78)	122	3.5	0.82
Sheep scab is a disease that makes my life more difficult	120	3.5	1.20
I feel ashamed when my flock has sheep scab	118	3.3	0.97
I get frustrated with having to live with sheep scab in my flock	117	3.5	0.94
Having sheep scab in my flock can make me feel miserable.	118	3.5	0.98
