# Supplementary material for: Behavioural Factors Influencing the Intention to Adopt Sheep Scab Control Measures in Northern Ireland
Source: Animals (Basel). 2024 Mar 15;14(6):912. doi: 10.3390/ani14060912 (PMC10967487; doi:10.3390/ani14060912)
Supplement: Supplementary file 1 [file animals-14-00912-s001.zip › animals-2861013-supplementary.pdf]

Supplementary Materials

To check the consistency of the statistically significant variables in our model, we also ran our analyses, excluding the non-significant variables in the models. All the variables remained statistically significant except for the emotional effect variable for the intention to pay for blood test (in the model without the socioeconomic variables) but with the same negative sign. This reflects the initial marginal level of statistical significance (P<0.1) of the variable. The results obtained are presented below.

Table S1. Estimates of OLOGIT model for eTPB constructs with only statistically significant variables.

	Intention to undertake blood test				Intention to pay for blood test			
Variables	Coef.	Std. Err.	%	%StdX	Coef.	Std. Err.	%	%StdX
Attitude (ATT)	0.185**	0.078	20.3	52.6	0.216***	0.076	24.1	63.8
Perceived behavioural control (PBC)	-0.200**	0.100	-18.1	-31.1				
Emotional effect	-0.298**	0.121	-25.8	-36.8	-0.164	0.111	-15.1	-22.3

Note: % is the percentage change in odds for a unit increase in the explanatory variable; %StdX is the percentage change in odds for a standard deviation change in the explanatory variable; single, double, and triple asterisks (*, **, ***) indicate significance at the 10%, 5%, and 1% levels, respectively.

Table S2. Estimates of OLOGIT model with adjustment for socio-demographic factors with only statistically significant variables.

	Undertake blood testing				Willingness to pay for blood testing			
Variables	Coef.	Std. Err.	%	%StdX	Coef.	Std. Err.	%	%StdX
Attitude (ATT)	0.275***	0.087	31.6	87.3	0.245***	0.078	27.8	75.0
Emotional effect	-0.204*	0.123	-18.5	-27.0				
BDG membership	1.158***	0.414	218.4	74.0				
Age (Less than 55 years					0.849**	0.347	133.8	53.1
Less than or 5 GCSEs or equivalent	1.163**	0.509	220.1	69.6				
A Level-Higher education or equivalent	1.545***	0.562	368.7	93.0				
Degree level or higher	1.349**	0.538	285.5	76.3				

Note: % is the percentage change in odds for unit increase in the explanatory variable; %StdX is the percentage change in odds for a standard deviation change in the explanatory variable; single, double, and triple asterisks (*, **, ***) indicate significance at the 10%, 5%, and 1% level, respectively.
